# Comparison of access-hole filling materials for screw retained implant prostheses: 12-month in vivo study

**DOI:** 10.1186/s40729-017-0076-4

**Published:** 2017-05-05

**Authors:** Rémy Tanimura, Shiro Suzuki

**Affiliations:** 1 8, place du Général Catroux, 75017 Paris, France; 2 Department of Clinical Community and Sciences, University of Alabama at Birmingham School of Dentistry, 1919 7th Avenue South, Birmingham, AL 35294-0007 USA

**Keywords:** Dental implant, Screw-retained, Access-hole, Wear, 4-META

## Abstract

**Background:**

Screw retained implant prostheses seem to be an efficient restorative method to prevent peri-implantitis caused by cement excess around the abutment. The drawback of the screw-retained prostheses is the difficulty to realize an efficient access-hole filling functionally and aesthetically. Up to now, few in vitro and in vivo studies were reported in the literature. The aim of this study was to evaluate clinical performances of two direct filling materials through a period of 12 months.

**Methods:**

To pursue a previous in vitro evaluation, this in vivo 12 months prospective study followed up and compared the access-hole filling integrity of a modified 4-META (4-methacryloxyethyl trimellitate anhydride)/MMA-TBB (methyl methacrylate-tri-*n*-butyl borane) – based resin (M4M) and a photo-polymerizing nano-hybrid composite resin (CR).

Twenty-eight access-holes were filled with both materials respectively, then impressions and intra-oral photographs were taken at *T* = 0, *T* = 1 M (month), 3, 6, and 12 M. The access-hole surface measurement and the margin analysis (depth and angle) were carried out. The VAS (visual analogue scale) value on marginal discoloration and integrity at the baseline *T* = 0 and *T* = 12 M was recorded.

**Results:**

The mean values of the surface areas changes from *T* = 1 to *T* = 12 M were 83.3 ± 11.5% for group CR and 77.1 ± 13.1% for group M4M, respectively. (Mann-Whitney test *p* < 0.05, *p* = 0.046). The mean marginal depth at *T* = 12 M for group CR were 141.2 ± 125.5 μm and 132.1 ± 107.8 μm for the group M4M, respectively. (Mann-Whitney test *p* > 0.05, *p* = 0.58). The mean values of the angle formed at the margin (*T* = 12 M) were for group CR 39.5 ± 19.4° and 28.2 ± 17.2° for group M4M, respectively (Mann-Whitney test *p* < 0.0001). The photographical analysis by VAS values showed no significant difference between CR and M4M groups (Mann-Whitney test *p* > 0.05, *p* = 0.848).

**Conclusions:**

Based on intra- and extra-oral evaluations with the limitation, both CR and M4M combined with a ceramic primer are indicated as promising materials to fill the access-hole. Further long-term investigation is necessary to confirm this finding.

## Background

The retention of implant-supported prostheses is provided by the use of a screw or cement. Recently, it was demonstrated that cement-retained prostheses had a higher rate of technical and biological complications [1], despite a better passive fit than the screw-retained restorations [2]. The CAD/CAM development of the implant-supported prostheses allows a better passively fit with screw-retained prostheses [3], and the development of the mechanics of screws reduced screw-loosening complications [4]. Screw retained prostheses can be retrievable and seem to be an efficient restorative method to prevent peri-implantitis caused by cement excess around the abutment [5, 6]. Nevertheless, these restorations have some disadvantages due to the presence of an access-hole opening that can alter the occlusal morphology and reduce the fracture resistance of the ceramic [7, 8]. It is reported that the integrity of the access-hole filling is in relation with the ceramic fracture resistance [9]. The esthetic outcome of the access hole filling is also influenced by the marginal integrity and the long-term stability of the filling material [10, 11].

An in vitro evaluation of a modified 4-META (4-methacryloxyethyl trimellitate anhydride)/MMA-TBB (methyl methacrylate-tri-*n*-butyl borane) – based resin (M4M) was conducted to compare the wear behavior to a photo-polymerizing nano-hybrid composite resin (CR), and the results were quite promising [12].

The aim of this in vivo study was to compare the access-hole filling integrity of two different filling materials, M4M and CR, during 12 months. The null hypothesis was that superficial and marginal deterioration of M4M and CR would not be significantly different.

## Methods

A total of 60 access-holes in 14 patients (5 male and 9 female) aging from 34 to 69 were restored and observed during 12 months. All subjects were informed about the study, and their written consent to participate in the study was taken.

The materials used in this study are presented in Table 1. They include a phosphoric acid monomer ceramic primer (CP): UCP (Super-Bond Universal Ceramic Primer, Sun Medical, Moriyama, Japan), a photo-polymerizing nanohybrid composite (FS): (Fantasista, shade A2, Sun Medical) and its accompanying photo-polymerizing bonding agent (BA):(Hybrid Bond, Sun Medical) and a modified 4-META/TBB-MMA resin (M4M) :(Bondfill SB, Sun Medical).

**Table 1:** ᅟ

Materials	Product names	Batch numbers	Manufacturer
Ceramic primer	Super-bond UCP	FX1	Sun Medical
Composite	Fantasista	GF11	Sun Medical
Bonding agent	Hybrid bond	FS1/GL1	Sun Medical
Adhesive composite	Bondfill SB	FT2/FS2/FS12	Sun Medical

Access-hole fillings were divided into CR and M4M groups.

Prior to the filling, the bottom of the access holes was filled with a PTFE (polytetrafluoroethylene) film (GEB SAS, Roissy CDG, France) to protect the screw. The thickness of this protective layer was approximately 2 mm.

### CR group

CP was applied and immediately air blown. BA was applied for 20 s, air blown for 5 s and then photo-polymerized using a polymerizing unit (Kerr Demi™plus, KavoKerr Group, Washington DC, USA) for 3–5 s. FS was placed by an incremental technique with less than 1 mm thickness for each layer until covering the top of the access hole. Each layer was photo-polymerized for 20 s. The occlusal adjustment was carried out with a diamond bur (Komet 368EF.204.023, Gebr. Brasseler GmbH & Co. KG, Lemgo, Germany), and the polishing was performed using a series of silicone polishers (Komet 9400, 9401, and 9402) with a 5000-rpm speed under water irrigation.

### M4M group

**Fig. 1:**
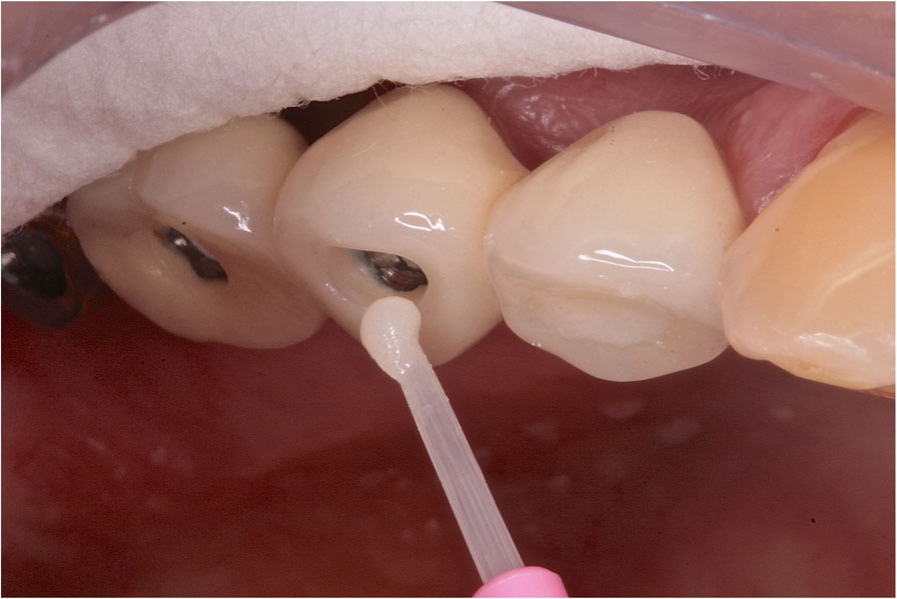
Brush-dip technique

CP was applied and air blown. Then, the base liquid of M4M was activated by adding the TBB initiator (3:1 ratio), and a powder/liquid mixture was applied, using a brush-dip technique until the filling of the access hole was completed (Fig. 1). The resin was left for 10 min to complete auto-polymerization. The occlusal adjustment was carried out with the same manner, and the polishing was performed using silicone polishers (Komet 9557, 9553) under the same condition.

Patients who received a metal framed ceramic screw retained implant crown or bridge were included in this study. These access holes were delimited only by ceramic. Those with metal surface exposure were excluded from this study. Patients with edentulous arch or section, full or partial denture as antagonists were excluded from the study. During the evaluation period, 2 patients (male) dropped out from this study. One access-hole (AMB/22) with an atypical shape (non-circular) causing a noticeable overfilling of the M4M was excluded from this study. Finally, 12 patients with a total of 56 access-holes were examined (28 access holes for both groups, CR and M4M). For each patient, access-holes were randomly divided in right and left sides to get equivalent numbers for both materials. All the fillings (CR and M4M groups) were performed by a single operator.

**Fig. 2:**
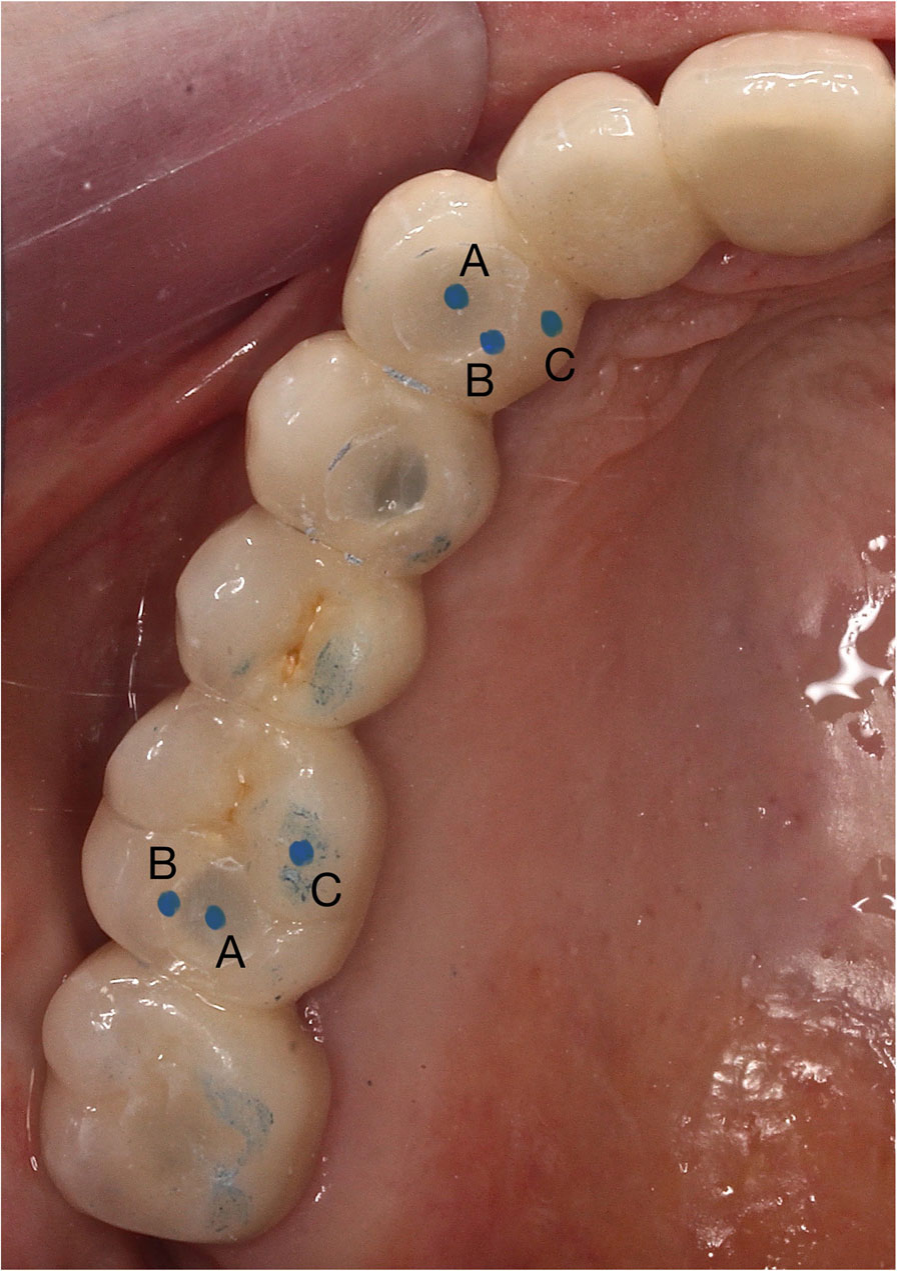
Occlusal contact point

For each access-hole, the contact patterns of antagonistic cusp in the centric occlusion were recorded intra-orally. They included; A: contact in the center, B: contact on the border of the access-hole and C: no contact close to the border (Fig. 2). At the time of the restoration (*T* = 0), followed by 1, 3, 6, and 12 months (*T* = 1, 3, 6, and 12 M), impressions were taken with polysiloxane impression materials (President, Light Body, (lot: G01914), Soft Putty (lot: G08568), Coltène/Whaledent AG, Switzerland) in a double mixed method. Epoxy casts (Devcon ET, Lot: 350402, ITW PP&F, Japan) were made from these impressions, and the fillings were examined using a motorized digital microscope (DSX510, Olympus KeyMed Ltd, USA) with a 1 μm accuracy under ×30 digital magnification.

For each clinical recall (*T* = 0 to *T* = 12 M), intra-oral photographs of the access hole fillings were taken.

### Surface areas changes of access-hole filling

**Fig. 3:**
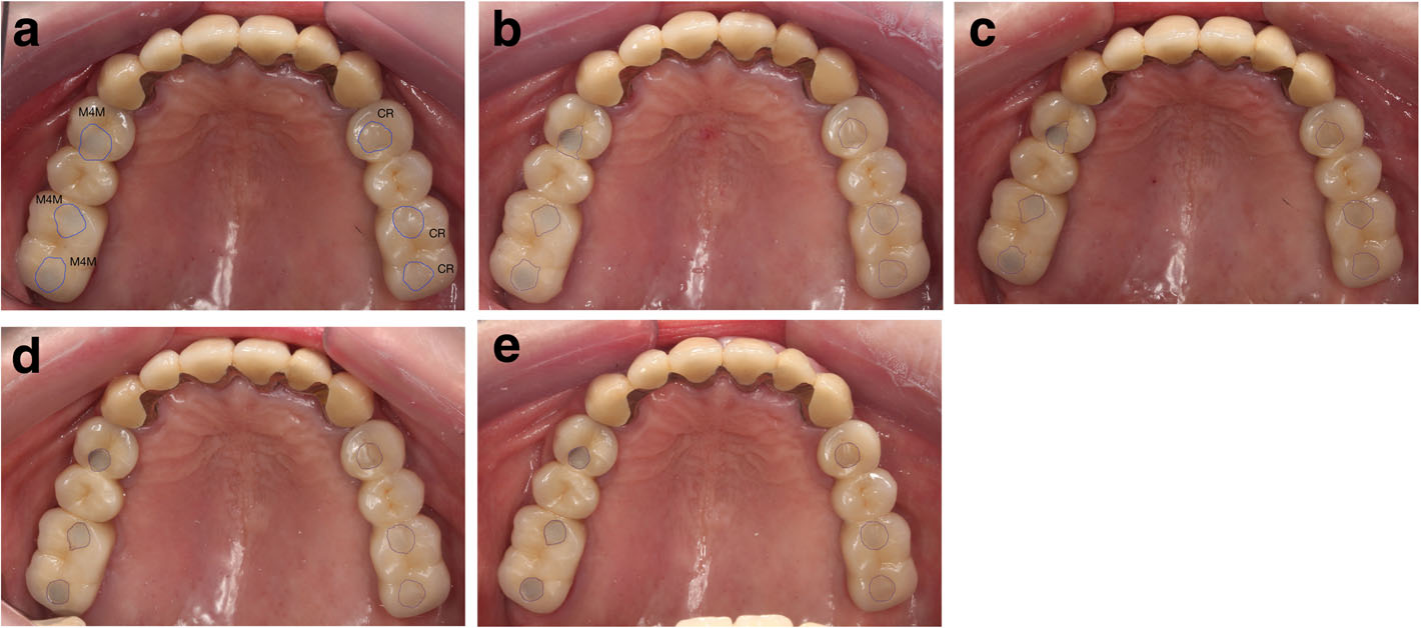
**a**–**e** (Filling surface changes): **a** (ROG, *T* = 0). **b** (ROG, *T* = 1 M). **c** (ROG, *T* = 3 M). **d** (ROG, *T* = 6 M). **e** (ROG, *T* = 12 M)

The surface areas of access holes could not be compared as the configuration of access-holes varied in each case. It was therefore necessary to compare longitudinal changes of the surface areas. At the time of *T* = 0, margin of the filling could not be clearly identified, thus, the measurement was fixed to begin at *T* = 1 M. For each sample, from *T* = 1 M to *T* = 12 M, the surface areas of access-hole filling were measured perpendicular to the vertical surface of the hole using the same digital microscope. The surface at *T* = 1 M was considered as 100%, and compared with the *T* = 3, 6, and 12 M of identical filling (Fig. 3a–e).

### Marginal analysis (depth and angle)

**Fig. 4:**
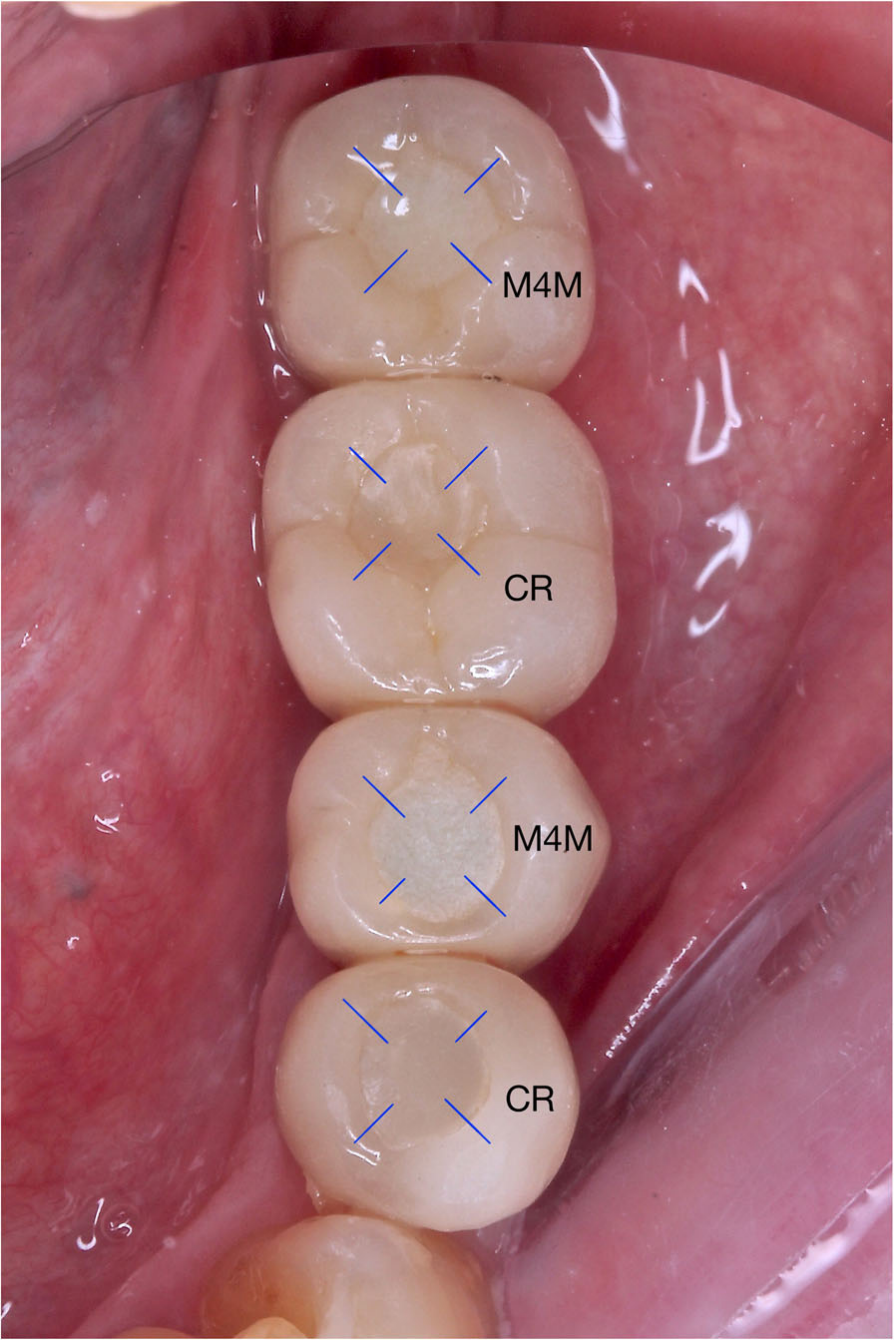
Margin depth measurement localization (example: TRA, *T* = 12 M)

**Fig. 5:**
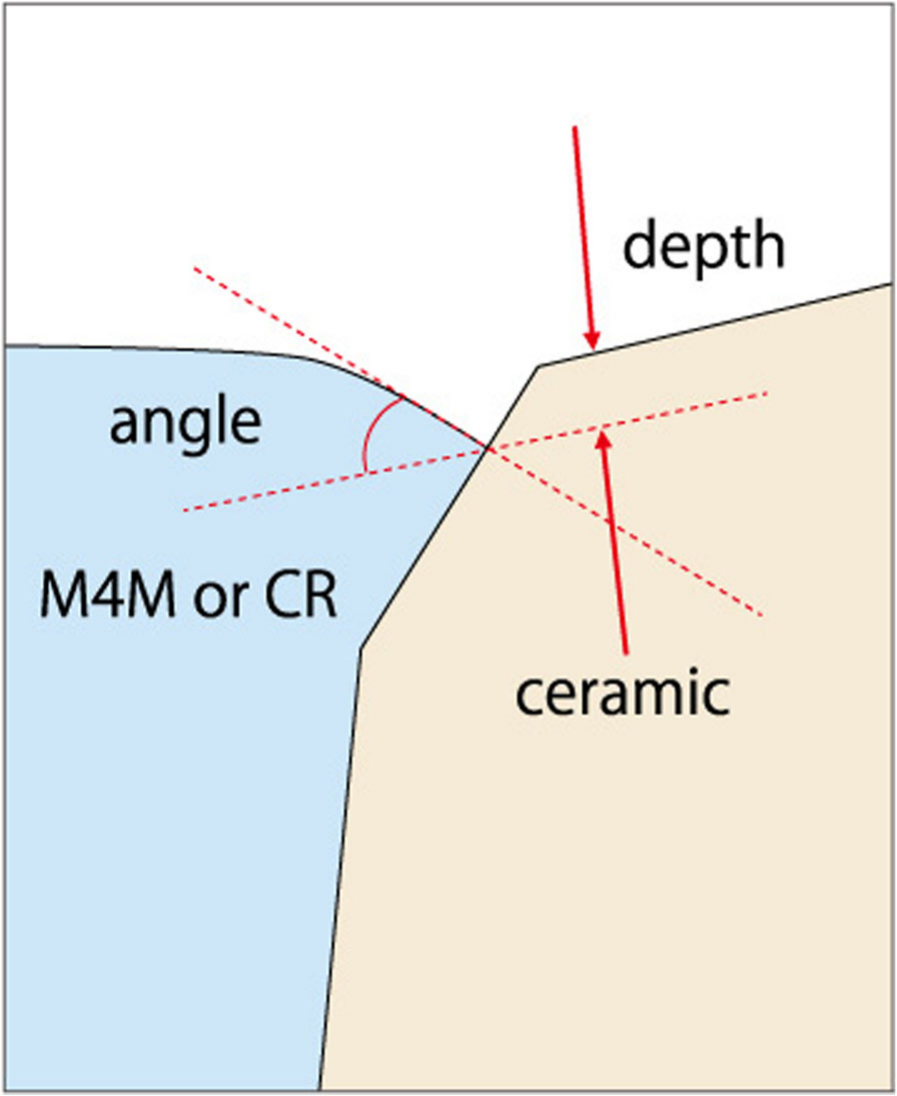
Depth and angle at the margin

The marginal depth of the access-hole filling at *T* = 0, *T* = 1 M, *T* = 3 M, and *T* = 6 M could not be defined successfully because of the overfilling phenomenon which disrupted the measurement of marginal gap depth and angle (Fig. 3a–e). Only the marginal depth and angle at *T* = 12 M could be measured with the same digital microscope, and the mean value for each group was calculated. Each access-hole was divided into four areas including mesio-buccal, disto-buccal, mesio-palatal and disto-palatal surfaces (Fig. 4) to measure the distance of marginal discrepancy at resin/ceramic interface with a 1 μm accuracy (Fig. 5). With this value, a “marginal discrepancy pattern” could be extrapolated for each group (Fig. 7a, b).

### Photographical analysis

For each access hole, an occlusal photograph was taken at the time of *T* = 0, *T* = 1 M, *T* = 3 M, *T* = 6 M, and *T* = 12 M, respectively. The marginal integrity was evaluated and recorded at the baseline and 12 months, according to an esthetical scale VAS (visual analogue scale) described by Weininger et al. [11]. Results were recorded by the authors and summarized in Table 2.

**Table 2:** Aesthetical Outcomes at T = 12 M (VAS Score)

Patients	Position CR			Patients	Position M4M		
		*T* = 0	*T* = 12			*T* = 0	*T* = 12
AMB	13	10	7	AMB	23	10	7
	14	6	5		24	8	3
	16	8	8		26	8	5
	17	10	10		27	8	5
ROG	24	10	7	ROG	14	10	6
	26	10	10		16	10	7
	27	10	10		17	10	7
NEU	11	4	3	NEU	21	6	5
	13	8	7		23	6	5
	16	8	7		25	10	10
POU	36	10	9	POU	27	10	10
TRA	34	10	8	TRA	35	10	10
	36	10	10		37	10	10
PHU	21	4	3	PHU	11	6	5
	25	10	5		14	8	8
	26	8	5		16	10	9
	27	8	5		17	10	9
ORT	15	10	7	ORT	14	10	10
					16	10	7
SUG	45	10	8	SUG	35	8	8
	47	10	10		37	8	8
KAI	46	8	6				
FRA	45	8	8	FRA	25	10	10
	46	10	10		26	8	8
	47	10	10		27	6	6
SHI	37	6	4	SHI	16	8	6
HAS	35	10	10	HAS	45	10	10
	36	8	8		46	8	8
	37	8	8		47	8	8
Average		8.64	7.43	Average		8.71	7.50
SD		1.81	2.23	SD		1.46	2.00

(*T* = 0)–(*T* = 12 M): CR 1.21 (SD = 1.37), M4M 1.21 (SD = 1.52) Mann-Whitney *p* > 0.05 (*p* = 0.848)

0: Filling completely dislodged

1: Filling partially dislodged

2: Access hole appearance and poor masking

3: 40% masking, with a marginal staining

4: 40% masking, no marginal staining

5: 60% masking, with a marginal staining

6: 60% masking, no marginal staining

7: 80% masking, with a marginal staining

8: 80% masking, no marginal staining

9: Total masking, with a marginal staining

10: Total masking, no marginal staining

## Results

Among the 56 access holes, no filling was dislodged during 12 months, and no complaint was registered from the patients regarding functional and aesthetical aspects.

### Surface areas changes

**Fig. 6:**
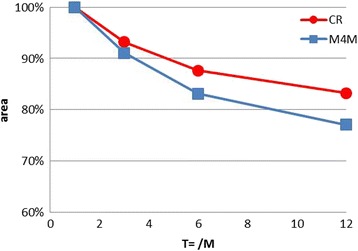
Access-hole filling surface areas measurement, average

The results for surface areas changes of access-hole fillings at respective intervals were summarized in Table 3 and Fig. 6. The mean values of the change from *T* = 1 M to *T* = 12 M were 77.1 ± 13.1% for group M4M and 83.3 ± 11.5% for group CR, respectively. They were not statistically different (Mann-Whitney test *p* < 0.05, *p* = 0.046).

**Table 3:** Surface areas changes of access-hole filling. Unit: %

	*T* = 1 M	*T* = 3 M	*T* = 6 M	*T* = 12 M
CR	100	93.2	87.6	83.3
M4M	100	91.1	83.2	77.1

### Contact mode and the surface areas changes

The contact distribution was as follows; *A* = 2, *B* = 14, and *C* = 40. The access-hole distributions were 24(CR) and 24(M4M) for the premolar/molar region, 4(CR) and 4(M4M) for the incisor/canine region, respectively. Among 16 access-holes, 11 of M4M and 5 of CR presented “B” (14) or “A” (2) occlusal contact mode. The average of the 11 (M4M) changes of the filling surface area at the time of *T* = 12 M was 77.1%. By pure coincidence, this mean value was identical to that of the 28 (M4M). For the 5 CR, the filling surface at *T* = 12 M was 85.3%. The average of the 28 CR was 83.3%.

### Marginal discrepancy: depth and angle analysis

The marginal depths of each access-hole (4 points) at *T* = 12 M were measured. The mean values for group CR were 141.2 ± 125.5 μm and 132.1 ± 107.8 μm for the group M4M, respectively. There was no significant difference between groups CR and M4M (Mann-Whitney test *p* > 0.05, *p* = 0.58).

**Fig. 7:**
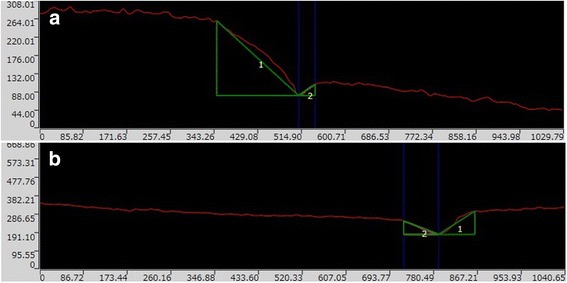
**a**, **b** (The marginal discrepancy pattern for group CR and M4M). **a** Group CR (1: Ceramic surface, 2: CR surface) Units of the axis are in μm. **b** Group M4M (1: Ceramic surface, 2: M4M surface) Units of the axis are in μm

The mean values of the angle at *T* = 12 M for group CR were 39.5 ± 19.4° and 28.2 ± 17.2° for group M4M, respectively. There was a significant difference (Mann-Whitney test *p* < 0.0001) between the groups CR and M4M. The marginal discrepancy patterns for both groups CR and M4M were different and shown in Fig. 7a, b.

### Photographical analysis

Discoloration of the filling material as well as the marginal integrity was recorded at *T* = 0 and *T* = 12 M (Table 2). For the CR group, the mean VAS values were 8.64 at *T* = 0 and 7.43 at *T* = 12 M. The difference was 1.21 ± 1.37. For the M4M group, the mean VAS values were 8.71 at *T* = 0 and 7.50 at *T* = 12 M. The difference was 1.21 ± 1.52. No significant difference was found in CR and M4M groups (Mann-Whitney test *p* > 0.05, *p* = 0.848).

## Discussion

Nowadays, implant screw-retained prosthesis becomes a popular mode of implant supra-structure restoration. Cement retained implant restoration has issues including irretrievability and difficulty of controlling the cement excess beyond the abutment joint. The cement excess can be a major cause of peri-implantitis [13–15]. Screw-retained implant restoration has also some disadvantages including the difficulty to get a right positioning of the access-hole compatible with a suitable aesthetic appearance and the aesthetic result of the access-hole restoration [16]. Inclined abutment or angling the screw channel can be an option to ameliorate the aesthetical outcomes [17, 18]. Moving the access hole to an occlusal or palatal/lingual zone would increase the indication of screw retained implant restoration. The development of the computer-assisted implant surgery increases the use of the screw-retained prosthesis compared to the cement retained method [19, 20]. Nevertheless, the integrity of the filling of the access hole is necessary to preserve to occlusal function and the aesthetic outcomes. Moreover, the access hole filling contributes to reinforce the surrounding ceramic [9].

In this context, access-hole filling is a common clinical procedure, but studies are quite limited in the literature. Photo-polymerizing resin composites have been widely used to fill the ceramic access holes, but unfortunately, the prognoses were less favorable [21–23]. The use of an efficient ceramic primer (CP) containing phosphoric acid monomer is mandatory to ensure durable adhesion to the feldspathic ceramic surface [24, 25]. In the previous in vitro study [12], ceramic access-hole specimens filled without ceramic pre-treatment showed no bond strength regardless of the use of the bonding agent. For this reason, in this in vivo study, the CP was used for groups CR and M4M.

Ceramic surfaces can be modified by silicate blasting or acid etching procedures to achieve a reliable adhesion [26, 27]. Hydrofluoric acid treatment has also been recommended but demands considerable precautions [28]. Clinically, decontamination by ultrasonic device during these procedures cannot be done easily [29]. It is also important to reduce clinical steps for access-hole filling.

The in vivo evaluation is quite different from that of the in vitro analysis. In the previous study, all the specimens were calibrated to a flat surface with 2.5 mm diameter. In present study, each access hole had a different dimension and configuration. The filling surfaces were often ø3 to 4 mm in diameter. Access-holes located in the occlusal groove area or in the inclined cusp surface induced an overfilling (Fig. 3a–e).

In clinical situations, masticatory stresses are quite different from the standard three-body generalized wear test [30]. Factors associating with wear processes including occlusal force, direction and speed of food excursion are individually different according to the location of the access-hole. Therefore, in this study, both filling groups were arranged in a symmetrical random way. For these reasons, the evaluation of changes in surface areas, marginal wear pattern, and marginal depth of fillings were selected.

### Surface areas changes

The surface areas of filling were reduced with time due to the wear of overflowed material that occurs systematically in a clinical situation. At the baseline (*T* = 0), M4M group presented the overfilling more frequently compared to the CR group possibly due to the low viscosity of the material during the setting time. M4M is much more fluid than CR, and the setting time duration is longer. The surface reduction by the occlusal wear seems to be smaller when the filling surface comes closer to the vertical inner wall of the access hole (Figs. 3a–e and 6). This particularity is the main difference with the previous in vitro study [12].

This surface reduction due to the wear might have a threshold value in relation with the material thickness at the margin and the toughness of the material regarding the compressive strength against the occlusal loading. A long-term analysis should be conducted to determine if this surface reduction will decrease with time.

Focusing on numbers of access-hole showing a disappearance of the overfilling in an early period (up to T = 3 M), groups CR and M4M exhibited 82% (23/28) and 39.3% (11/28), respectively (Table 4). This can be explained by the differences of mechanical properties of both materials. The elastic moduli of M4M and CR are 1.9 and 7.9 MPa, respectively. Flexural strength of M4M is 66 MPa which is comparable to that of an acrylic resin (60 MPa) and almost half compared to CR (115 MPa) [31, 32]. The low flexural strength of M4M possibly absorbed the occlusal stresses, and the overfilling stayed longer compared to CR.

**Table 4:** Disappearance of the overfilling. Unit: %

	*T* = 1 M	*T* = 3 M	*T* = 6 M	*T* = 12 M	No disappearance
CR	53.5% (15/28)	28.5% (8/28)	7.1% (2/28)	3.6% (1/28)	7.1% (2/28)
M4M	28.6% (8/28)	10.7% (3/28)	28.5% (8/28)	28.5% (8/28)	3.6% (1/28)

According to the results of contact mode and the surface areas changes, it was suggested that there were no correlations among the occlusal contact modes and the surface areas reduction up to 12 months in vivo.

### Marginal depth analysis

As mentioned in the previous study [12], the margin of M4M group had a better adhesion to the surrounding access hole ceramic and no gap formation was observed between the cavity and the filling material, due to the specific polymerization mode (TBB initiator). The bond strengths on the glazed feldspathic ceramic were 7.6 ± 2.2 MPa for group CR, and 8.6 ± 1.0 MPa for group M4M. In some cases, adhesive failure mode was detected at the margin with group CR. The polymerization shrinkage of the CR might have deteriorated the adhesion quality at the margin [33].

The elastic modulus of a filling material and its flexural strength influence the wear resistance at the marginal zone [31]. The difference of the polymerization mode modifies the stress distribution at the marginal areas. M4M is polymerized with the TBB auto-polymerizing catalyst; therefore it shows low shrinkage at the marginal surface and possesses an advantage of minimizing gap formation [34]. The marginal areas of CR might receive stress concentration, and subsequent micro-gap formation possibly occurred due to polymerization shrinkage during the photo-polymerization. The wear values of filling material itself influence the wear pattern. The 3-body wear values are 1.5 mm [3] for the acrylic resin, 0.9 mm [3] for the M4M and 0.3 mm [3] for the CR. The toothbrush wear test shows the same tendency [34]. Although, CR contains greater amounts of filler (TMPT fillers) compared to M4M (CR:71.5 wt%, M4M:<10 wt%), M4M has an aptitude for lower brittleness compared to CR as its matrix consists of acrylic resin which possesses flexibility [35].

### Photographical analysis

The analysis for the ratio of access-hole with or without marginal staining at the time of *T* = 12 M showed that the results in group CR (12/16) and in group M4M (12/16) were the same. For this reason, the marginal discrepancy pattern, different in both groups did not influence the aesthetical result. The occlusal contact point (A, B, or C) was compared to the marginal staining rate. Among the access-holes with A or B contact point, 6 out of 16 presented the marginal staining at the time of *T* = 12 M. For C contact point, 18 out of 40 presented the marginal staining at the time of *T* = 12 M. No correlation was admitted between the occlusal contact mode and the marginal staining.

An opaque composite resin for the CR group and an opaquer powder for the M4M group might improve the esthetical result and hide the metal frame or the prosthetic screw in the access-hole [36, 37].

Some studies analyzed the effectiveness of a ceramic inlay to restore an access hole [38]. Above the screw, a channel of 3 to 4 mm is needed to achieve this technique and in some clinical situations, there is less than 2 mm. Despite the excellent aesthetical results, with ceramic inlay technique, crown or bridge retrieval is harder and generates an additional cost. The poor clinical result reported with the composite resin filling (control group) might be the fact that a ceramic primer and a bonding agent were not used.

## Conclusions

Within the limitation of the study, it was concluded that:

- The null-hypothesis “superficial and marginal deterioration of M4M and CR would not be significantly different” was accepted.
- The M4M (modified 4-META/MMA-TBB resin) and CR (composite resin) combined with a ceramic primer showed comparable characteristics (marginal integrity and wear behavior) in an access hole filling.
- The esthetical evaluation (VAS scale) showed that there were no significant differences between group M4M and CR.
- After 12 months, the pattern of marginal wear showed a difference, but both materials clinically worked out successfully.

Further long-term investigation is necessary to confirm these findings.
